# Outcomes of patients with systemic lupus erythematosus treated with belimumab: a post hoc efficacy analysis of five phase III clinical trials by British Isles Lupus Assessment Group-based Combined Lupus Assessment criteria

**DOI:** 10.1136/rmdopen-2025-005444

**Published:** 2025-04-23

**Authors:** Ioannis Parodis, Julius Lindblom, Leonardo Palazzo, Nursen Cetrez, Shereen Oon, Henri Ala, Ronald F van Vollenhoven, Eric Morand, Adrian Levitsky, Mandana Nikpour

**Affiliations:** 1 Division of Rheumatology, Department of Medicine Solna, Karolinska Institutet, Karolinska University Hospital, and Center for Molecular Medicine (CMM), Stockholm, Sweden; 2 Department of Rheumatology, Faculty of Medicine and Health, Örebro University, Örebro, Sweden; 3 Department of Rheumatology, St Vincent’s Hospital Melbourne, Melbourne, Victoria, Australia; 4 Department of Medicine, The University of Melbourne at St Vincent’s Hospital, Melbourne, Victoria, Australia; 5 Department of Rheumatology and Clinical Immunology, Amsterdam University Medical Centers, Amsterdam, The Netherlands; 6 Centre for Inflammatory Diseases, Monash University, Melbourne, Victoria, Australia; 7 School of Public Health and Sydney MSK Research Flagship Centre, The University of Sydney, Sydney, New South Wales, Australia; 8 Department of Rheumatology, Royal Prince Alfred Hospital, Sydney, New South Wales, Australia

**Keywords:** Systemic Lupus Erythematosus, Lupus Erythematosus, Systemic, Biological Therapy, Outcome Assessment, Health Care

## Abstract

**Objectives:**

To determine belimumab efficacy assessed using the British Isles Lupus Assessment Group (BILAG)-based Combined Lupus Assessment (BICLA) in patients with systemic lupus erythematosus (SLE) from phase III belimumab randomised controlled trials (RCTs).

**Methods:**

A post hoc analysis was carried out on five RCTs in active adult SLE: four with intravenous (BLISS-52, BLISS-76, BLISS-NEA, EMBRACE) and one with subcutaneous belimumab (BLISS-SC). The 52-week landmark assessments were analysed across trials. Treatment response was defined according to BICLA criteria (BILAG improvement; no worsening of disease activity based on BILAG and Systemic Lupus Erythematosus Disease Activity Index-2K; no deterioration in Physician’s Global Assessment ≥0.3 (scale: 0–3); no treatment failure).

**Results:**

A total of 3086 patients received belimumab (n=1869) or placebo (n=1217). BICLA response frequencies at week 52 were greater with belimumab vs placebo in BLISS-52 (OR (95% CI): 1.49 (1.05–2.12); p=0.024), BLISS-NEA (1.62 (1.12–2.33); p=0.010) and BLISS-SC (1.89 (1.39–2.57); p<0.001). A highly significant difference was observed in the pooled population (1.47 (1.25–1.72); p<0.001; adjusted for trial variance). Belimumab yielded greater BICLA response frequencies than placebo irrespective of baseline glucocorticoid dose (>7.5 or ≤7.5 mg/day of a prednisone equivalent), in patients with baseline SLEDAI-2K≥10 and in patients with positive anti-double-stranded (ds)DNA and/or low C3/C4 levels at baseline. Belimumab combined with anti-malarials yielded greater frequency of BICLA response attainment.

**Conclusions:**

In this analysis of five RCTs evaluating belimumab in SLE, belimumab conferred superiority over placebo to yield BICLA response in the overall study population and in subgroups of patients with high global or serological activity at baseline. The benefit of belimumab was more prominent when combined with anti-malarials.

**WHAT IS ALREADY KNOWN ON THIS TOPIC**

- Different clinical responder indices, for example, systemic lupus erythematosus (SLE) Responder Index 4 (SRI-4) and BILAG-based Combined Lupus Assessment (BICLA), are commonly used outcome measures in SLE clinical trials, while it is unclear which are preferable for use as a primary endpoint. It is therefore important to perform post hoc analyses with another clinical responder index than the original one chosen to assess consistency with the original results.

**WHAT THIS STUDY ADDS**

- The original trial results of five key phase III SLE trials assessing belimumab efficacy with SRI-4 were herein validated in a pooled analysis with an additional key clinical responder index, BICLA, thus further demonstrating the efficacy of belimumab in treating SLE.
- BICLA response frequencies to belimumab were superior to placebo in patients with baseline Systemic Lupus Erythematosus Disease Activity Index (SLEDAI)-2K≥10 but not in those with SLEDAI-2K<10 and in patients with positive anti-double-stranded (ds)DNA antibody levels and/or low C3 and/or C4 levels at baseline but not in patients negative for anti-dsDNA and normal/high C3 and C4 levels, corroborating the notion that patients with high SLE disease activity and serologically active patients are more likely to benefit from belimumab.

**HOW THIS STUDY MIGHT AFFECT RESEARCH, PRACTICE OR POLICY**

- The results from this post hoc analysis of foundational phase III SLE belimumab trials have important implications for clinical practice, as they substantiate the clinical efficacy of belimumab through an additional widely used responder index of SLE. These findings may inform future trial design, while also supporting the use of belimumab in the management of SLE, in accordance with current recommendations.

## Introduction

 Systemic lupus erythematosus (SLE) is a chronic autoimmune disease with a multitude of manifestations affecting various organs and systems leading to inflammation, damage and dysfunction. It is characterised by a heterogeneous clinical presentation, variable disease course, significant morbidities including unpredictable flares and reduced health-related quality of life and loss of life expectancy.^1 2^ Tenets to the management of SLE include the control of disease by targeted treatment to remission or low disease activity, preventing organ damage and improving patients’ health-related quality of life.^3^,^7^

While there have been treatment advances beyond traditional immunosuppressive anti-rheumatic agents in SLE, disease activity assessment remains challenging due to the complex and diverse nature of disease manifestations. Several disease activity indices have been developed and validated for SLE, such as the Systemic Lupus Erythematosus Disease Activity Index (SLEDAI) and the British Isles Lupus Assessment Group (BILAG) index.^8^,^10^ However, these indices have limitations, such as the SLEDAI lacking sensitivity to change and not including disease manifestations, such as haemolytic anaemia and pulmonary haemorrhage, or the BILAG being complex, time-consuming and thus difficult to apply widely in clinical practice.^11^ The Physician’s Global Assessment (PGA) serves as a comprehensive disease activity marker, while it has sizeable inter-rater variability likely due to unstandardised scoring including use of both pointed anchored and continuous centimetric scales and thus limited reliability.^12 13^ Recognising the stand-alone limitations of instruments like the SLEDAI, BILAG and PGA, composite responder indices have been developed including the SLE Responder Index 4 (SRI-4)^14^ and the BILAG-based Combined Lupus Assessment (BICLA),^15^ which are commonly used outcome measures and endpoints in SLE clinical trials. While SRI-4 reflects response based on the presence or absence of a lupus manifestation rather than the severity of a manifestation as responder classification is mainly conditioned by the SLEDAI, BICLA enables incremental response assessment. Given that differences in trial outcomes may depend on which index is chosen as the primary endpoint,^16 17^ it is important to carry out additional analyses with another key responder index.

Belimumab is a monoclonal antibody binding to a cytokine that is considered important in SLE pathogenesis, that is, B-Lymphocyte Stimulator (BLyS), also known as B cell activating factor belonging to the tumour necrosis factor family (BAFF). It was approved for treating SLE following its demonstration of reducing disease activity through several randomised clinical trials (RCTs),^18^,^22^ and post-hoc analyses further demonstrated its ability to induce remission and low disease activity.^23^,^25^ The purpose of this study was to determine belimumab efficacy assessed using BICLA in patients with SLE included in the phase III belimumab RCTs, in which SRI-4 outcomes were originally assessed.

## Methods

### Study population

We conducted a post hoc analysis using data from five clinical trials, that is, BLISS-52 (intravenous belimumab; NCT00424476; n=865),^18^ BLISS-76 (intravenous belimumab; NCT00410384; n=819),^19^ BLISS-NEA (intravenous belimumab in Northeast Asia; NCT01345253; n=677),^20^ BLISS-SC (subcutaneous (SC) belimumab; NCT01484496; n=836)^22^ and EMBRACE (IV belimumab in SLE patients of African ancestry; NCT01632241; n=448).^21^ We analysed the initial 52 weeks of follow-up across these trials. Our study included data from 3086 patients who received either the approved belimumab dose (10 mg/kg/month intravenous or 200 mg/week SC; n=1869) or placebo (n=1217) in addition to standard therapy.

Subgroup analyses were carried out to investigate data from patients with the following baseline characteristics: SLEDAI 2000 (SLEDAI-2K)^8^ score ≥10 or SLEDAI-2K<10; positive or negative anti-double-stranded (ds)DNA antibody status; positive anti-dsDNA antibody status and/or low levels of complement component 3 (C3) and/or C4 or negative anti-dsDNA antibody status and normal/high C3/C4 levels; and a daily glucocorticoid dose of prednisone or a prednisone equivalent >7.5 mg or ≤7.5 mg.

We obtained data from the RCTs from GlaxoSmithKline (Uxbridge, UK) through the Clinical Study Data Request (CSDR) consortium. Prior to original enrolment in the trials, written informed consent was obtained from all study participants. The trial protocols were subject to review and approval by regional ethics review boards at all participating centres, and they adhered to the ethical principles of the Declaration of Helsinki. The protocol for the present post-hoc analysis was approved by the Swedish Ethical Review Authority (registration number: 2019–05498).

### Clinical definitions

The BICLA definition requires BILAG-2004 improvement in all affected domains at baseline (A domain scores to B, C or D and B domain scores to C or D) and no worsening of disease activity based on other organ systems of BILAG-2004 (defined as ≥1 new A domain score(s) or ≥2 new B domain scores); no increase in the SLEDAI-2K score from baseline; no numeric increase in the PGA score ≥0.3 points from baseline (range: 0–3); and no treatment failure.^15 26^ Treatment response in this post-hoc analysis was defined as a modified BICLA definition utilising the Classic BILAG^10^ instead of BILAG-2004 since this was the version of BILAG that was used in the assessed trials.^18^,^2127^ Where BICLA response was not achieved based on at least one criterion, this was considered non-response, irrespective of missing data in other criteria. Where one or more criteria were met but key data were missing preventing confirmation of all criteria, the response was classified as ‘not available’. Patients who had no BILAG A or B domain scores at baseline—and were therefore ineligible for improvement according to the BICLA definition—were excluded from the analysis.

Organ damage was determined using the Systemic Lupus International Collaborating Clinics/American College of Rheumatology Damage Index (SDI).^28^

### Serological markers

Conventional serological markers at baseline were examined for their potential role as determinants of BICLA response; the selection of markers was based on data availability. These included the presence of anti-dsDNA antibodies (≥30.0 IU/mL); anti-Smith (Sm) antibodies (≥15.0 IU/mL); anti-ribonucleoprotein (RNP) antibodies (≥25.0 IU/mL); anti-ribosomal P antibodies (≥12.0 IU/mL in BLISS-52; ≥12.4 IU/mL in BLISS-76; ≥12.5 IU/mL in BLISS-SC); anti-phospholipid antibodies (presence of any), including anti-cardiolipin (aCL) IgA (≥10.0 IU/mL in BLISS-52 and BLISS-76; ≥11.0 IU/mL in BLISS-NEA, BLISS-SC and EMBRACE), IgG (≥10.0 IU/mL in BLISS-52 and BLISS-76; ≥14.0 IU/mL in BLISS-NEA, BLISS-SC and EMBRACE) or IgM antibodies (≥10.0 IU/mL in BLISS-52 and BLISS-76; ≥12.0 IU/mL in BLISS-NEA, BLISS-SC and EMBRACE); anti-β_2_-glycoprotein I (β_2_-GPI) IgA, IgG or IgM antibodies (≥21.0 IU/mL for all Ig isotypes); or lupus anti-coagulant (LAC), defined as a diluted Russell viper venom time >45 s in BLISS-SC and as lupus anti-coagulant-sensitive aPTT>41 s in EMBRACE, based on laboratory-specific reference ranges. LAC data were not available in BLISS-52, BLISS-76 or BLISS-NEA. We also investigated low levels of C3 (<90.0 mg/dL), low levels of C4 (<16.0 mg/dL in BLISS-52 and BLISS-76; <10.0 mg/dL in BLISS-NEA, BLISS-SC and EMBRACE) and serum BLyS/BAFF levels.

### Statistical analysis

Descriptive statistics are reported as numbers (percentage) or means (SD), and medians (IQR) are indicated in case of non-normal distributions. For crude comparisons between belimumab and placebo, the non-parametric Mann-Whitney U test was used for continuous variables, and the Pearson’s *χ*^2^ test was used for binomial variables. Comparisons between belimumab and placebo were also made using logistic regression analysis adjusting for trial variance. Variables in the multivariable logistic regression analysis evaluating predictors of BICLA response included variables deemed clinically important (age, sex and self-reported ancestry) and variables with sufficient available values (<5% missing data) that reached statistical significance (p<0.050) in the initial models. ORs, 95% CIs and *p* values were calculated from week 4 through week 52. Differences yielding *p* values <0.05 were deemed statistically significant. Analyses were performed with the *tableone* and *stats* R packages, using the R Statistical Software V.4.2.1 (R Foundation for Statistical Computing, Vienna, Austria).

### Patient and public involvement

The public was not involved in the design, conduct, reporting or dissemination plans of this research. Patient research partners from the Swedish Rheumatism Association were involved in the design of the study and the dissemination plans.

## Results

Of a total of 3086 patients who received the approved dosage regimens of belimumab or placebo, 1869 received belimumab (intravenous or SC) and 1217 received placebo. Among these, 2802 had calculable BICLA response status at least once during follow-up, comprising 1693 patients in the belimumab group and 1109 in the placebo group. Patient characteristics did not differ between the belimumab and placebo groups, with the exception of ancestry (table 1).

**Table 1:** Patient demographics and clinical features at baseline

	All patients(n=2802)	Belimumab + ST(n=1693)	Placebo + ST(n=1109)
Demographics			
Age* (years); mean (SD)	37.3 (11.7)	37.1 (11.4)	37.7 (12.2)
Female sex; n (%)	2641 (94)	1598 (94)	1043 (94)
Race†; n (%)			
Asian	933 (33)	589 (35)	344 (31)
Black/African American	602 (22)	382 (23)	220 (20)
Indigenous American‡	285 (10)	153 (9)	132 (12)
White/Caucasian	982 (35)	569 (34)	413 (37)
Clinical and serological features			
SLE disease duration (years); mean (SD)§	6.5 (6.4); n=2801	6.4 (6.3); n=1692	6.6 (6.5)
SLEDAI-2K; mean (SD)	10.6 (3.6)	10.7 (3.6)	10.5 (3.6)
<10; n (%)	1077 (38)	642 (38)	435 (39)
≥10; n (%)	1725 (62)	1051 (62)	674 (61)
PGA score; mean (SD)	1.5 (0.5); n=2797	1.5 (0.5); n=1690	1.5 (0.5); n=1107
0 to 1; n (%)	249 (9); n=2797	157 (9); n=1690	92 (8); n=1107
>1 to 2.5; n (%)	2508 (90); n=2797	1508 (89); n=1690	1000 (90); n=1107
>2.5; n (%)	40 (1); n=2797	25 (2); n=1690	15 (1); n=1107
SDI score; median (IQR)	0.0 (0.0–1.0)	0.0 (0.0–1.0)	0.0 (0.0–1.0)
SDI score≥1; n (%)	424 (38.2)	561 (33.1)	985 (35.2)
Anti-dsDNA positive**¶**; n (%)	1967 (70)	1204 (71)	763 (69)
Low** C3 and/or C4; n (%)	1584 (57)	966 (57)	618 (56)
Low** C3 and/or C4 and anti-dsDNA positive**¶**; n (%)	1350 (48)	835 (49)	515 (46)
Medications			
Concomitant SLE medication; n (%)			
GC	2458 (88)	1482 (88)	976 (88)
AMA	1951 (70)	1177 (70)	774 (70)
Immunosuppressants	1457 (52)	865 (51)	592 (53)
GC and AMA and immunosuppressants	836 (30)	513 (30)	323 (29)
GC (prednisone equivalent) dose (mg/day); mean (SD)	11.8 (9.2)	11.9 (9.4)	11.6 (9.0)
GC dose (mg/day) category; n (%)			
0	344 (12)	211 (13)	133 (12)
>0 to ≤7.5	713 (25)	418 (25)	295 (27)
>7.5	1745 (62)	1064 (63)	681 (61)

Data are presented as numbers (percentage) or means (standard deviationSD). In case of non-normal distributions, the median (interquartile rangeIQR) is indicated. In case of missing values, numbers of patients with available data are indicated. Anti-malarial agents included hydroxychloroquine, chloroquine, and, in a minority of cases, mepacrine, mepacrine hydrochloride, and quinine sulphate.

* Age was imputed when full date of birth was not available. who fit more than one race category were counted under the individual race category according to the minority rule as well as the multiracial category.

† Patients who fit more than one race category were counted under the individual race category according to the minority rule as well as the multiracial category.Alaska Native or American Indian from North, South or Central America.

‡ Alaska Native or American Indian from North, South or Central America.Disease duration was defined as (screening date/treatment start date − SLE diagnosis date)/365.25.

§ Disease duration was defined as (screening date/treatment start date − SLE diagnosis date+1)/365.25.cut-off for anti-dsDNA positivity:.

¶ Cut-off for anti-dsDNA positivity: ≥30 IU/mL.Low C3 cut-off:; low C4 cut-off: in BLISS-SC, BLISS-NEA and EMBRACE, and in BLISS-76 and BLISS-52.

** Low C3 cut-off, <90 mg/dL; low C4 cut-off, <10 mg/dL in BLISS-SC, BLISS-NEA and EMBRACE; and <16 mg/dL in BLISS-76 and BLISS-52.Age was imputed when full date of birth was not available.

AMA, anti-malarial agents; anti-dsDNA, anti-double-stranded DNA antibodies; C3, complement component 3; C4, complement component 4; GC, glucocorticoid; PGA, Physician’s Global Assessment; SDI, Systemic Lupus International Collaborating Clinics (SLICC)/American College of Rheumatology (ACR) Damage Index; SLE, systemic lupus erythematosus; SLEDAI-2K, SLE Disease Activity Index 2000; ST, standard therapy.

### British Isles Lupus Assessment Group-based Combined Lupus Assessment (BICLA) at week 52

**Figure 1:**
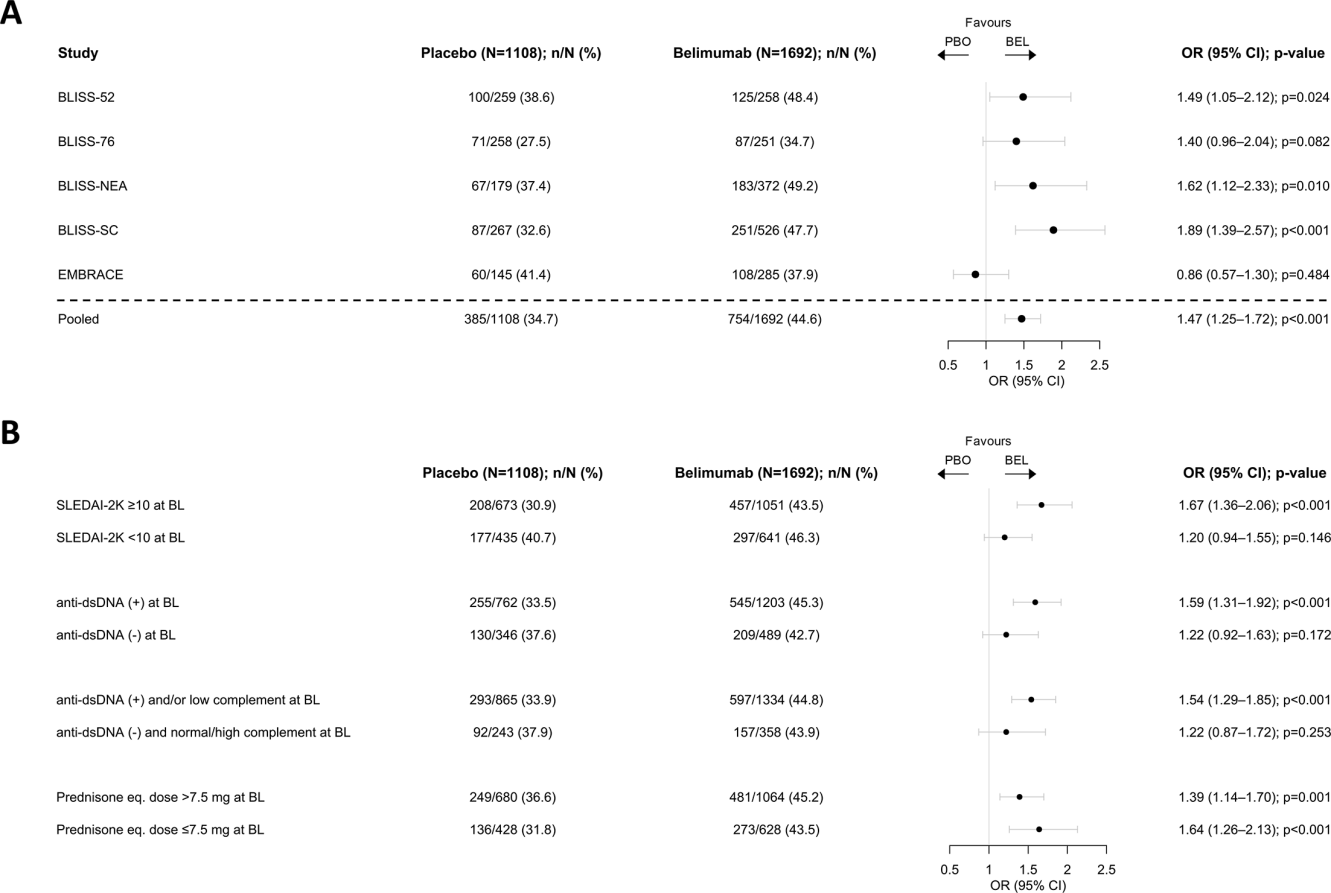
Pooled analysis of five phase III randomised controlled trials assessing belimumab efficacy: BICLA response assessment at 52 weeks of follow-up. (**A**) Forest plot illustrating results from logistic regression analysis of BICLA response attainment in belimumab-treated patients vs placebo recipients on individual trial level and analysis of pooled data from all trials, after adjusting for trial variance. (**B**) Forest plot illustrating results from pooled analysis in patient subgroups of interest based on baseline clinical and serological data. (+), positive levels; (-), negative levels; anti-dsDNA, anti-double-stranded DNA antibodies; BEL, belimumab; BICLA, British Isles Lupus Assessment Group-based Combined Lupus Assessment; BL, baseline; eq., equivalent; PBO, placebo; SLEDAI-2K, Systemic Lupus Erythematosus Disease Activity Index 2000.

Of the 3086 participants who were eligible for participation in this study, 2800 (91%) had calculable BICLA response status at week 52 and were analysed on an individual trial level (n=belimumab vs placebo, respectively) across five SLE clinical trials: BLISS-52 (n=258 vs 259); BLISS-76 (n=251 vs 258); BLISS-NEA (n=372 vs 179); BLISS-SC (n=526 vs 267); and EMBRACE (n=285 vs 145). Significant differences in BICLA response frequencies at week 52 were observed in favour of belimumab versus placebo in BLISS-52 (OR: 1.49; 95% CI 1.05 to 2.12; p=0.024), BLISS-NEA (OR: 1.62; 95% CI 1.12 to 2.33; p=0.010) and BLISS-SC (OR: 1.89; 95% CI 1.39 to 2.57; p<0.001). A numerical but statistically non-significant difference in favour of belimumab was observed in BLISS-76 (OR: 1.40; 95% CI 0.96 to 2.04; p=0.082). No difference was observed in EMBRACE (OR: 0.86; 95% CI 0.57 to 1.30; p=0.484). A highly significant difference in BICLA response at week 52 in favour of belimumab versus placebo was observed in analysis of pooled data from all trials, after adjusting for trial variance (OR: 1.47; 95% CI 1.25 to 1.72; p<0.001; figure 1A).

In pooled subgroup analyses, belimumab was superior to placebo in inducing BICLA response among patients with baseline SLEDAI-2K≥10 (OR: 1.67; 95% CI 1.36 to 2.06; p<0.001) but not among those with baseline SLEDAI-2K<10 (OR: 1.20; 95% CI 0.94 to 1.55; p=0.146), among patients with positive anti-dsDNA antibody levels at baseline (OR: 1.59; 95% CI 1.31 to 1.92; p<0.001) but not among patients with negative anti-dsDNA antibody levels at baseline (OR: 1.22; 95% CI 0.92 to 1.63; p=0.172) and among patients with positive anti-dsDNA antibody levels and/or low complement (C3 and/or C4) levels at baseline (OR: 1.54; 95% CI 1.29 to 1.85; p<0.001) but not among those negative for anti-dsDNA and with normal/high C3 and C4 levels at baseline (OR: 1.22; 95% CI 0.87 to 1.72; p=0.253). Belimumab was superior to placebo in inducing BICLA response regardless of baseline glucocorticoid dose (figure 1B).

### Longitudinal British Isles Lupus Assessment Group-based Combined Lupus Assessment (BICLA) response assessment from week 4 to week 52

**Figure 2:**
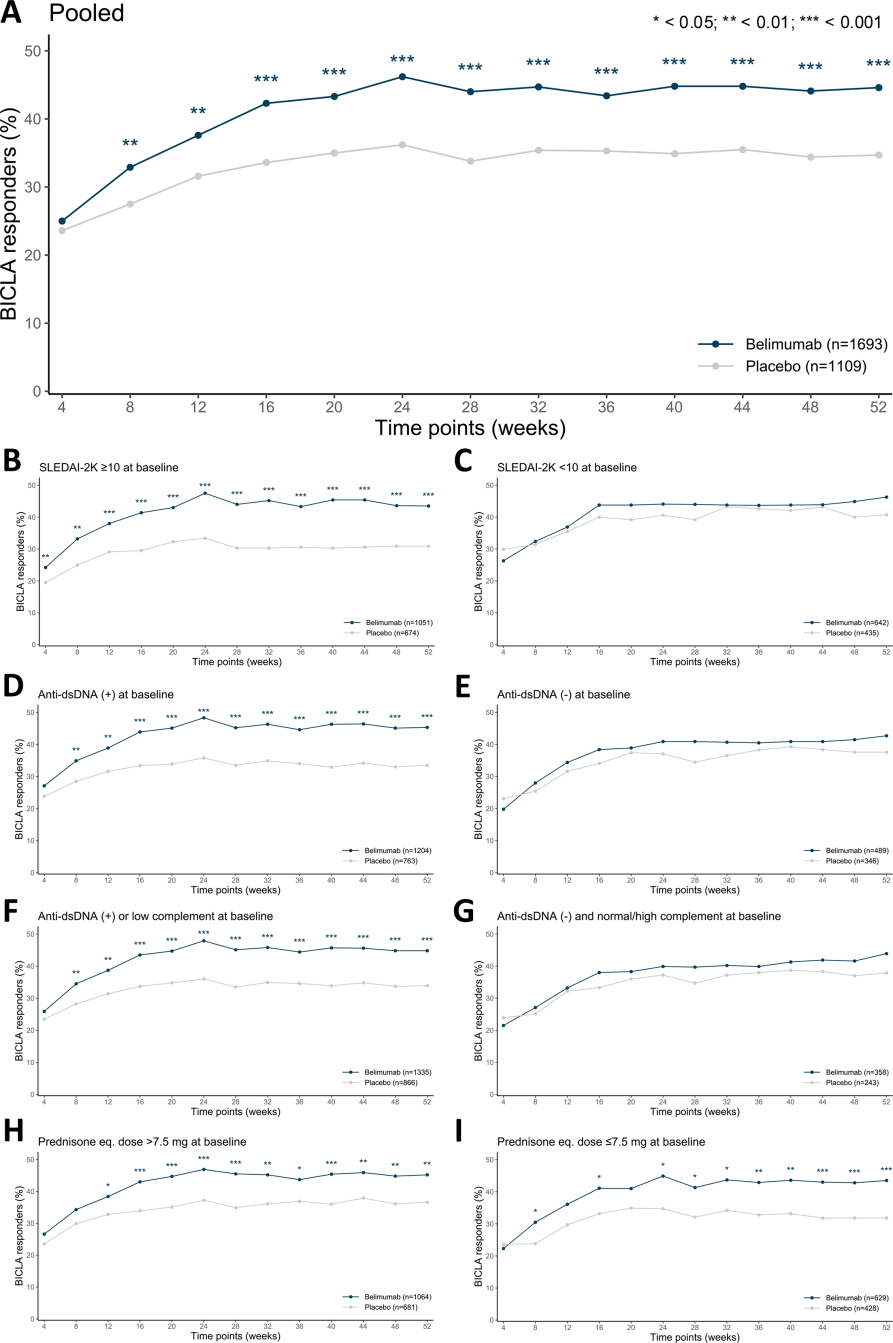
BICLA responders, all time points up to 52 weeks of follow-up. Graphs illustrating results from logistic regression analysis adjusting for trial variance with BLISS-52 and BLISS-76 pooled as a reference. Plotted are frequencies of BICLA response from week 4 through week 52 (**A**) in patients with SLE treated with belimumab (blue lines) versus placebo (grey lines) in the pooled study population and (B–I) in subgroups of patients based on baseline clinical and serological data. Dots denote frequencies and asterisks indicate statistically significant *p* values. (+), positive levels; (-), negative levels; anti-dsDNA, anti-double-stranded DNA antibodies; BICLA, British Isles Lupus Assessment Group (BILAG)-based Combined Lupus Assessment; eq., equivalent; SLEDAI-2K, Systemic Lupus Erythematosus Disease Activity Index 2000.

Pooled analyses from all trials by specific time points from week 4 to week 52 indicated a significantly higher proportion of BICLA responders among belimumab-treated patients than among patients in the placebo group starting at week 8 (557 (32.9%) of 1693 patients in the belimumab group vs 305 (27.5%) of 1108 patients in the placebo group; OR: 1.27; 95% CI 1.07 to 1.50; p=0.006) and with sustainedly greater responder frequencies in favour of belimumab versus placebo at all 4-week assessments thereafter up to week 52 (754 (44.6%) of 1692 patients in the belimumab group vs 385 (34.7%) of 1108 patients in the placebo group; OR: 1.47; 95% CI 1.25 to 1.72; p<0.001; figure 2A; online supplemental table S1).

Subgroup analyses by specific time points indicated more pronounced differences between the belimumab and placebo groups for the subgroups denoting high disease activity at baseline by SLEDAI-2K and serology compared with their opposing subgroups. Among patients with baseline SLEDAI-2K≥10, a statistically significant difference in BICLA response attainment in favour of belimumab was seen as early as week 4 (254 (24.2%) of 1051 patients in the belimumab group vs 131 (19.5) of 673 patients in the placebo group; OR: 1.34; 95% CI 1.05 to 1.71; p=0.017) and maintained throughout the 52-week study period (figure 2A; online supplemental table S2). Similarly, statistically significant differences in favour of belimumab were observed as early as week 8 among patients with positive anti-dsDNA antibody levels at baseline (420 (34.9%) of 1204 patients in the belimumab group vs 217 (28.5) of 762 patients in the placebo group; OR: 1.31; 95% CI 1.07 to 1.60; p=0.009), as well as among patients with positive anti-dsDNA antibody levels and/or low C3 and/or C4 levels at baseline (460 (34.5%) of 1335 patients in the belimumab group vs 244 (28.2%) patients in the placebo group; OR: 1.30; 95% CI 1.08 to 1.57; p=0.006). In both subgroups, the statistical significance in the separation between belimumab and placebo was maintained throughout the study period (figure 2D,F; online supplemental tables S4 and S6). By contrast, in the subgroups of patients with SLEDAI-2K<10 at baseline (figure 2C; online supplemental table S3), patients negative for anti-dsDNA at baseline (figure 2E; online supplemental table S5) and patients negative for anti-dsDNA and normal/high C3 and C4 levels at baseline (figure 2G; online supplemental table S7), the differences between belimumab and placebo in BICLA response frequencies did not reach statistical significance. Belimumab was superior to placebo in inducing BICLA response regardless of baseline glucocorticoid dose at most post-baseline assessments (figure 2H,I; online supplemental tables S8 and S9). Details regarding the results of subgroup logistic regression analyses are provided in figure 2B–I and online supplemental tables S2–S9.

### Influence of concomitant treatment with anti-malarial agents on British Isles Lupus Assessment Group-based Combined Lupus Assessment (BICLA) response

**Figure 3:**
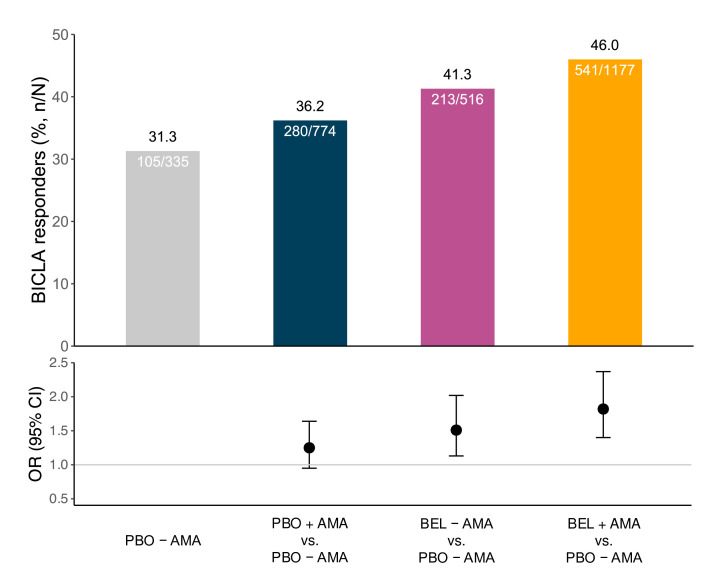
Influence of concomitant treatment with anti-malarial agents on BICLA response. Bar graphs (upper panel) and dot-and-whisker graph (lower panel) illustrating BICLA response frequencies at week 52 in patients treated with belimumab or placebo, with or without concomitant anti-malarials. AMA, anti-malarial agents; anti-dsDNA, anti-double-stranded DNA antibodies; BEL, belimumab; BICLA, British Isles Lupus Assessment Group (BILAG)-based Combined Lupus Assessment; PBO, placebo.

Concomitant treatment with anti-malarial agents (AMA) increased the proportion of BICLA responders both among belimumab-treated patients and placebo recipients (figure 3). Patients who received placebo and concomitant AMA had marginally greater frequencies of BICLA response compared with those who received placebo without AMA (36.2% vs 31.3%; OR: 1.25; 95% CI 0.95 to 1.64; p=0.114). The combination of belimumab with concomitant treatment of AMA yielded the greatest frequency of BICLA response at week 52, and a difference of 14.7% compared with placebo recipients not on AMA (46.0% vs 31.3%; OR: 1.82; 95% CI 1.40 to 2.37; p<0.001; figure 3; online supplemental table S10).

### Factors associated with British Isles Lupus Assessment Group-based Combined Lupus Assessment (BICLA) response at week 52

**Figure 4:**
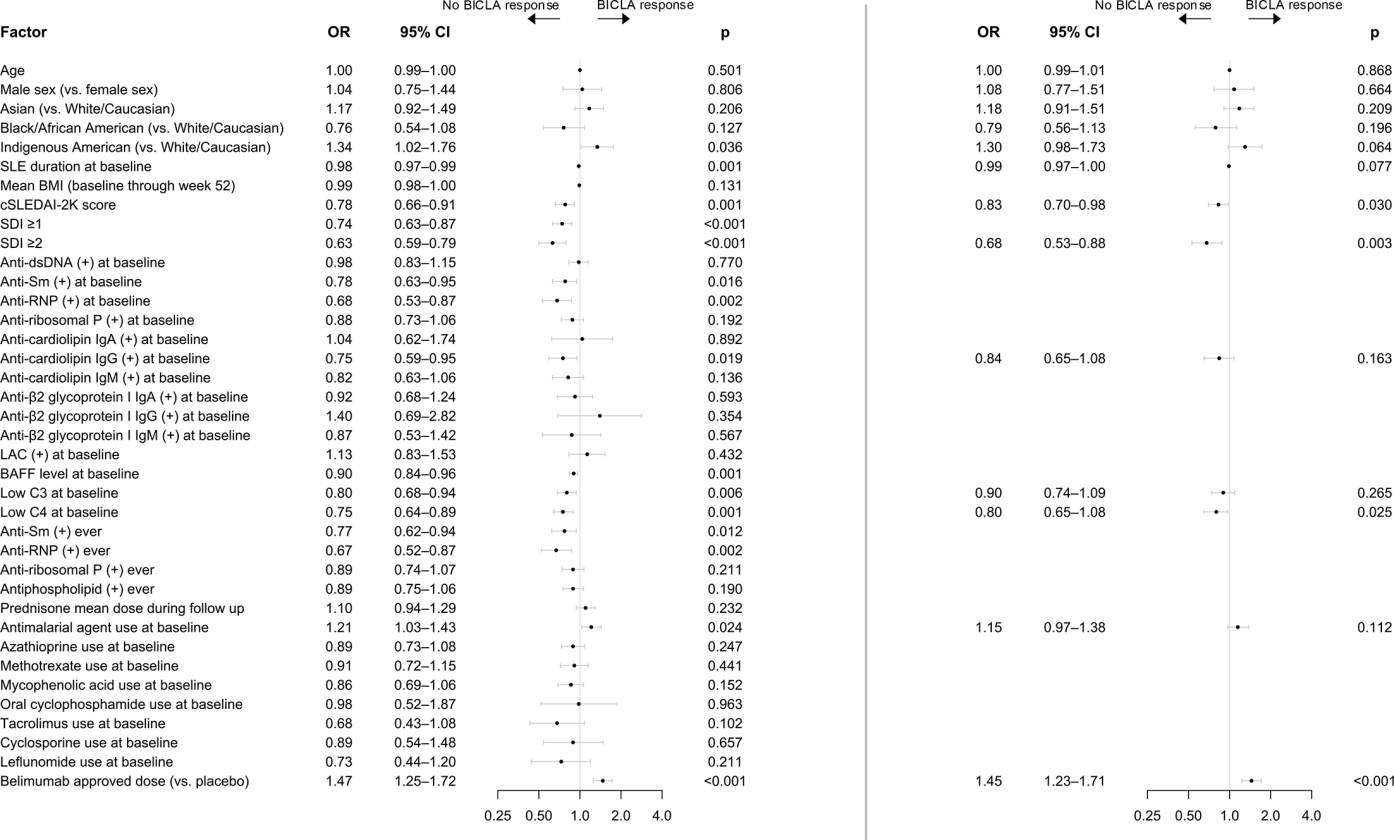
Predictors of BICLA response at 52 weeks of follow-up. Forest plot illustrating results from univariable (left) and multivariable (right) logistic regression analysis investigating predictors of BICLA response at 52 weeks of follow-up in the pooled study population. AMA, anti-malarial agents; anti-dsDNA: anti-double-stranded DNA antibodies; anti-RNP, anti-U1-ribonucleoprotein antibodies; anti-Sm, anti-Smith antibodies; BAFF, B cell activating factor belonging to the tumour necrosis factor family; BEL, belimumab; BICLA, British Isles Lupus Assessment Group (BILAG)-based Combined Lupus Assessment; BMI, body mass index; cSLEDAI-2K, clinical Systemic Lupus Erythematosus Disease Activity Index 2000; C3, complement component 3; C4, complement component 4; LAC, lupus anticoagulant; PBO, placebo; SDI, Systemic Lupus International Collaborating Clinics (SLICC)/American College of Rheumatology (ACR) Damage Index; SLE, systemic lupus erythematosus.

Following logistic regression analysis that was adjusted for trial variance, the Native American ancestry (OR: 1.34; 95% CI 1.02 to 1.76; p=0.036), concomitant treatment with AMA (OR: 1.21; 95% CI 1.03 to 1.43; p=0.024) and treatment with belimumab at approved doses (OR: 1.47; 95% CI 1.25 to 1.72; p<0.001) were positively associated with BICLA response at week 52. By contrast, a longer disease duration (OR: 0.98; 95% CI 0.97 to 0.99; p=0.001), increasing clinical SLEDAI-2K (cSLEDAI-2K, ie, SLEDAI-2K disregarding the serological descriptors) at baseline (OR: 0.78; 95% CI 0.66 to 0.91; p=0.001), SDI score≥1 (OR: 0.74; 95% CI 0.63 to 0.87; p<0.001) or ≥2 (OR: 0.63; 95% CI 0.50 to 0.79; p<0.001) at baseline, positive anti-Sm antibody levels at baseline (OR: 0.78; 95% CI 0.63 to 0.95; p=0.016) or at any measurement during the study follow-up (OR: 0.77; 95% CI 0.62 to 0.94; p=0.012), positive anti-RNP antibody levels at baseline (OR: 0.68; 95% CI 0.53 to 0.87; p=0.002) or at any measurement during the study follow-up (OR: 0.67; 95% CI 0.52 to 0.87; p=0.002), anti-aCL IgG positivity at baseline (OR: 0.75; 95% CI 0.59 to 0.95; p=0.019), elevated BLyS/BAFF levels at baseline (OR: 0.90; 95% CI 0.84 to 0.96; p=0.001), and low C3 (OR: 0.80; 95% CI 0.68 to 0.94; p=0.006) and C4 (OR: 0.75; 95% CI: 0.64 to 0.89; p=0.001) levels at baseline were all associated with a lower likelihood of BICLA response at week 52 (figure 4; online supplemental table S11).

In multivariable logistic regression analysis, treatment with belimumab at approved dose remained an independent predictor of BICLA response at week 52 (OR: 1.45; 95% CI 1.23 to 1.71; p<0.001), and increasing cSLEDAI-2K at baseline (OR: 0.83; 95% CI 0.70 to 0.98; p=0.030), baseline SDI score ≥2 (OR: 68; 95% CI 053 to 0.88; p=0.003) and low baseline levels of complement C4 (OR: 0.80; 95% CI 0.65 to 0.97; p=0.025) were independently associated with a lower likelihood of BICLA response at week 52. Results are illustrated in figure 4 and detailed in online supplemental table S12.

## Discussion

In this post hoc analysis of five foundational and explorative phase III RCTs assessing the clinical efficacy of belimumab in patients with SLE, belimumab conferred significantly greater benefit than placebo based on BICLA criteria for treatment response in a pooled analysis and particularly among patients with high disease activity (SLEDAI-2K≥10), anti-dsDNA positivity and/or low complement C3 and/or C4 levels at baseline. This analysis is therefore relevant to both clinical trial methodology and routine clinical decision-making. It confirms that two key SLE responder indices, SRI-4 and BICLA, demonstrate the clinical efficacy of belimumab, further substantiating its use in the management of patients with SLE.

Clinician-reported outcome measures of treatment response used in SLE have evolved over time, moving from the use of individual disease activity indices, such as SLEDAI,^29^ BILAG^10^ and PGA,^13^ to composite responder definitions that incorporate such indices, among which SRI-4^14^ and BICLA^15^ are the most widely used as primary endpoints in SLE clinical trials. Both SRI-4 and BICLA have strengths and limitations and may be considered complementary as they are stringent in different ways. While SRI-4 response assessment is primarily driven by improvement in the SLEDAI score,^14^ BICLA response assessment relies on improvement in the BILAG index.^15^ The SRI-4 response requires complete resolution of a manifestation in at least one SLEDAI organ domain, that is, improvement of ≥4 points.^14^ On the other hand, the BICLA definition of response necessitates improvement in all affected organ domains,^15^ ensuring a clinically meaningful response across all organ systems. By contrast, BICLA does not require complete resolution of a manifestation to be reached; partial improvement according to BILAG scoring may suffice.^15 30^ Divergent response evaluations using the SRI-4 or BICLA criteria have been observed in clinical trials,^31^ including the TULIP-1 trial of anifrolumab where superiority was observed for anifrolumab over placebo by BICLA criteria, but assessment using the SRI-4 failed to yield a statistically significant result.^16^ Collectively, while SRI-4 requires resolution of manifestations, BICLA captures partial improvements in all active organ systems without requiring full resolution, thus detecting more granular changes in disease activity; these differing requirements underscore their complementary nature in clinical trials. Our study documents the superiority of belimumab over placebo as per BICLA response, corroborating results from phase III RCTs that had used SRI-4 as the efficacy endpoint and thus strengthening the clinical importance of belimumab for the treatment of SLE.

It is important to note that BICLA response criteria are based on the BILAG-2004 index;^9^ however, in this study, we applied a modification of BICLA using the Classic BILAG index,^10^ as this was the version employed in the original RCTs. The BILAG-2004 index introduced some relevant additions, including two additional organ domains (ie, ophthalmic and gastrointestinal) in place of the vasculitis domain and new items better capturing mucocutaneous, renal and nervous system involvement.^9^ Importantly, the scoring of improvement changed in the BILAG-2004 version. In the Classic BILAG index, all improving items were scored as a C, regardless of initial scoring. This did not reflect the level of disease activity based on treatment needs for the more severe manifestations and was overcome in the BILAG-2004 index, where features contributing to an A score are scored with B when improving, as these features remain significant for treatment decisions.^32^ While it remains speculative whether differences between the BILAG-2004 and Classic BILAG scoring systems might have led to divergent results, we acknowledge the use of modified BICLA criteria based on the Classic BILAG as a limitation of our study.

In subgroup analyses, belimumab conferred significantly greater clinical benefit than placebo as per the BICLA response criteria in subgroups of patients that had high disease activity at baseline. This included patients with SLEDAI-2K score ≥10 and patients with positive anti-dsDNA antibody levels and/or low complement C3 and/or C4 levels. Our findings are consistent with a previous post hoc analysis of the BLISS-52 and BLISS-76 trials that investigated predictors of SRI-4 response to belimumab and identified a Safety Of Estrogens In Lupus Erythematosus National Assessment-SLEDAI^33^ score of 10 or greater, anti-dsDNA positivity, and low complement levels as baseline factors predictive of SRI-4 response at week 52.^34^ Similarly, evidence from a real-world setting also showed that high disease activity defined as a SLEDAI-2K score ≥10 was an independent predictor of SRI-4 response after up to 36 months from treatment commencement.^35^ Our analysis using BICLA as a response measure further validates that high SLE disease activity and/or serologically active disease are characteristics that denote enhanced likelihood to respond to belimumab therapy.

A finding of particular interest was that the proportion of BICLA responders was greater among patients with concomitant AMA treatment than among those without, both in the group of patients who were treated with belimumab and in patients who received placebo. This finding provides further support for the use of AMA in all patients with SLE unless contraindicated, as recommended.^36^ Moreover, although the association between AMA use and BICLA response was attenuated in fully adjusted multivariable regression analysis, the observed added efficacy of belimumab combined with AMA supports the notion of a synergistic effect between the two treatments. This complements findings from previous post hoc investigations in the same SLE population. In one study, higher frequencies of patient-reported EQ-5D full-health state were observed in one study among belimumab-treated patients concomitantly treated with AMA compared with belimumab without AMA.^37^ Another investigation showed that co-administration of belimumab and AMA yielded enhanced protection against the development of renal flares compared with belimumab without AMA.^38^ In yet another post hoc analysis of BLISS-52 and BLISS-76 data, intravenous belimumab 10 mg/kg induced significant decreases in IgG and IgA aCL levels over time only in the subgroup of patients who were concomitantly treated with AMA.^39^ Finally, a similar effect was shown for SC belimumab in a post hoc analysis of the BLISS-SC trial, which reported significantly greater decreases in IgM aCL and IgA anti-β_2_-GPI levels in the belimumab group compared with the placebo group only in the subgroup of patients concomitantly receiving AMA.^40^ Altogether, accumulating evidence supports the use of AMA in combination with belimumab for an increased likelihood of favourable responses to this biological therapy, which should be considered in future recommendations.

Some limitations of this study should be acknowledged. One limitation was the post hoc nature of our analysis. The original trials were not designed to use BICLA to assess efficacy, which may have limited the statistical power for showing differences between belimumab and placebo within individual trials. This was reflected by the lower number of patients being eligible for our post hoc analyses compared with the original trial populations. For this reason and given that subgroup analyses were predefined, no correction for multiple comparisons was applied. Nonetheless, this study demonstrated superiority of belimumab to placebo in inducing BICLA responses despite a smaller sample size eligible for analysis. Of note, we employed a modified BICLA definition based on the Classic BILAG index, as this was the version used in the original trials, which may limit direct comparability to studies employing BILAG-2004. Second, we acknowledge the selection bias imposed by the trial protocols, which excluded patients with severe central nervous system disease and/or severe active lupus nephritis, limiting the applicability of our findings for patients with these manifestations of SLE. Third, the clinical trial setting limits the generalisability of our findings to real-world clinical settings of SLE. Fourth, the proportion of patients treated with anti-malarials was lower than current real-world practice. This likely reflects variations in local standards of care and the time period during which the trials were conducted. However, this variation enabled us to discern the additive effect of anti-malarial use on BICLA responses. While adherence to anti-malarials could not be accounted for, any non-adherence is expected to have been balanced across trial arms and would likely have attenuated the observed additive effect. This is the first study to the authors’ knowledge to assess belimumab efficacy using BICLA in a large and diverse patient population of adults with active SLE from across the globe, with five RCTs as a basis. Furthermore, patients were followed up within the frame of controlled trial programmes, ensuring the reliability of the acquired data. Finally, while trial-specific differences exist, we accounted for trial variance in our analyses, and the large study population allowed us to perform multivariable regression analyses adjusting for a relatively large number of potential confounders, as well as predefined subgroup analyses, thereby mitigating concerns regarding heterogeneity.

In conclusion, using an additional key clinical responder index, BICLA, we validated the results from foundational trials originally assessing belimumab efficacy using SRI-4, thus corroborating the efficacy of belimumab in SLE. Important implications for clinical practice include the substantiation of the clinical efficacy of belimumab and support for its use as a treatment in the management of SLE, especially among patients with high disease activity or serologically active patients. Additionally, added benefits of belimumab were observed when used in combination with AMA.

## Supplementary material

10.1136/rmdopen-2025-005444 online supplemental file 1

## Data Availability

Data may be obtained from a third party and are not publicly available.
