# Supplementary material for: Outcomes of patients with systemic lupus erythematosus treated with belimumab: a post hoc efficacy analysis of five phase III clinical trials by British Isles Lupus Assessment Group-based Combined Lupus Assessment criteria
Source: RMD Open. 2025 Apr 23;11(2):e005444. doi: 10.1136/rmdopen-2025-005444 (PMC12020763; doi:10.1136/rmdopen-2025-005444)
Supplement: online supplemental file 1 [file rmdopen-11-2-s001.pdf]

Outcomes of patients with systemic lupus erythematosus treated with intravenous or subcutaneous belimumab: a post-hoc efficacy meta-analysis by BICLA criteria

Ioannis Parodis, Julius Lindblom, Leonardo Palazzo, Nursen Cetrez, Shereen Oon, Henri Ala, Ronald F. van Vollenhoven, Eric F. Morand, Adrian Levitsky, Mandana Nikpour

SUPPLEMENTAL MATERIAL

TABLE OF CONTENTS

Supplemental Table S1. BICLA response from week 4 through week 52 in patients with SLE treated with belimumab versus placebo in the pooled BLISS study population....3

Supplemental Table S2. BICLA response from week 4 through week 52 in patients with SLEDAI-2K ≥10 at baseline treated with belimumab versus placebo....4

Supplemental Table S3. BICLA response from week 4 through week 52 in patients with SLEDAI-2K <10 at baseline treated with belimumab versus placebo....5

Supplemental Table S4. BICLA response from week 4 through week 52 in patients with positive anti-dsDNA levels at baseline treated with belimumab versus placebo....6

Supplemental Table S5. BICLA response from week 4 through week 52 in patients negative for anti-dsDNA at baseline treated with belimumab versus placebo....7

Supplemental Table S6. BICLA response from week 4 through week 52 in patients with positive anti-dsDNA levels and/or low C3 and/or C4 levels at baseline treated with belimumab versus placebo....8

Supplemental Table S7. BICLA response from week 4 through week 52 in patients negative for anti-dsDNA and with normal/high C3 and C4 levels at baseline treated with belimumab versus placebo....9

Supplemental Table S8. BICLA response from week 4 through week 52 in patients with a prednisone equivalent dose >7.5 mg/day at baseline treated with belimumab versus placebo....10

Supplemental Table S9. BICLA response from week 4 through week 52 in patients with a prednisone equivalent dose ≤7.5 mg/day at baseline treated with belimumab versus placebo....11

Supplemental Table S10. Influence of concomitant antimalarial treatment on BICLA response at week 52....12

Supplemental Table S11. Factors associated with BICLA response at week 52 in patients with SLE in the pooled belimumab trial population....13

Supplemental Table S12. Factors associated with BICLA response at week 52 in patients with SLE in the pooled belimumab trial population by multivariable analysis....15

Supplemental Table S1. BICLA response from week 4 through week 52 in patients with SLE treated with belimumab versus placebo in the pooled BLISS study population.

	BEL; n (%); N	PBO; n (%); N	OR	95% CI	*p* value
**BICLA response**					
BICLA at week 4	423 (25.0); N=1693	261 (23.6); N=1108	1.08	0.90–1.29	0.404
BICLA at week 8	557 (32.9); N=1693	305 (27.5); N=1108	1.27	1.07–1.50	**0.006**
BICLA at week 12	635 (37.6); N=1691	350 (31.6); N=1107	1.29	1.09–1.52	**0.002**
BICLA at week 16	716 (42.3); N=1692	373 (33.6); N=1109	1.42	1.21–1.67	**<0.001**
BICLA at week 20	733 (43.3); N=1692	388 (35.0); N=1108	1.39	1.18–1.62	**<0.001**
BICLA at week 24	781 (46.2); N=1691	401 (36.2); N=1107	1.47	1.26–1.72	**<0.001**
BICLA at week 28	744 (44.0); N=1692	374 (33.8); N=1107	1.51	1.29–1.78	**<0.001**
BICLA at week 32	756 (44.7); N=1693	392 (35.4); N=1108	1.43	1.22–1.67	**<0.001**
BICLA at week 36	735 (43.4); N=1692	391 (35.3); N=1107	1.37	1.17–1.60	**<0.001**
BICLA at week 40	757 (44.8); N=1691	387 (34.9); N=1108	1.48	1.26–1.73	**<0.001**
BICLA at week 44	758 (44.8); N=1691	394 (35.5); N=1109	1.45	1.24–1.70	**<0.001**
BICLA at week 48	746 (44.1); N=1692	382 (34.4); N=1109	1.48	1.27–1.74	**<0.001**
BICLA at week 52	754 (44.6); N=1692	385 (34.7); N=1108	1.47	1.25–1.72	**<0.001**

Results from logistic regression analysis adjusting for trial variance with BLISS-52 and BLISS-76 pooled as a reference. Data are presented as the number of events (percentage), odds ratio (OR), 95% confidence interval (CI), and *p* value. Statistically significant *p* values are in bold.

BEL: belimumab; BICLA: British Isles Lupus Assessment Group (BILAG)-Based Composite Lupus Assessment; CI: confidence interval; OR: odds ratio; PBO: placebo.

Supplemental Table S2. BICLA response from week 4 through week 52 in patients with SLEDAI-2K ≥10 at baseline treated with belimumab versus placebo.

	BEL; n (%); N	PBO; n (%); N	OR	95% CI	*p* value
**BICLA response**					
BICLA at week 4	254 (24.2); N=1051	131 (19.5); N=673	1.34	1.05–1.71	**0.017**
BICLA at week 8	349 (33.2); N=1051	168 (25.0); N=673	1.45	1.17–1.81	**0.001**
BICLA at week 12	399 (38.0); N=1051	196 (29.1); N=673	1.47	1.19–1.82	**<0.001**
BICLA at week 16	435 (41.4); N=1051	199 (29.5); N=674	1.66	1.35–2.05	**<0.001**
BICLA at week 20	452 (43.0); N=1051	218 (32.3); N=674	1.56	1.27–1.92	**<0.001**
BICLA at week 24	499 (47.5); N=1051	225 (33.4); N=673	1.76	1.43–2.15	**<0.001**
BICLA at week 28	462 (44.0); N=1051	204 (30.3); N=673	1.76	1.43–2.16	**<0.001**
BICLA at week 32	475 (45.2); N=1051	204 (30.3); N=674	1.82	1.48–2.24	**<0.001**
BICLA at week 36	455 (43.3); N=1051	206 (30.6); N=673	1.68	1.37–2.07	**<0.001**
BICLA at week 40	477 (45.4); N=1051	204 (30.3); N=673	1.86	1.52–2.29	**<0.001**
BICLA at week 44	477 (45.4); N=1051	206 (30.6); N=674	1.85	1.50–2.28	**<0.001**
BICLA at week 48	458 (43.6); N=1051	208 (30.9); N=674	1.71	1.39–2.10	**<0.001**
BICLA at week 52	457 (43.5); N=1051	208 (30.9); N=673	1.67	1.36–2.06	**<0.001**

Results from logistic regression analysis, adjusting for trial variance with BLISS-52 and BLISS-76 pooled as a reference. Data are presented as the number of events (percentage), odds ratio (OR), 95% confidence interval (CI), and *p* value. Statistically significant *p* values are in bold.

BEL: belimumab; BICLA: British Isles Lupus Assessment Group (BILAG)-Based Composite Lupus Assessment; CI: confidence interval; OR: odds ratio; PBO: placebo; SLEDAI-2K: Systemic Lupus Erythematosus Disease Activity Index 2000.

Supplemental Table S3. BICLA response from week 4 through week 52 in patients with SLEDAI-2K <10 at baseline treated with belimumab versus placebo.

	BEL; n (%); N	PBO; n (%); N	OR	95% CI	*p* value
**BICLA response**					
BICLA at week 4	169 (26.3); N = 642	130 (29.9); N = 435	0.81	0.62–1.07	0.144
BICLA at week 8	208 (32.4); N = 642	137 (31.5); N = 435	1.01	0.77–1.31	0.961
BICLA at week 12	236 (36.9); N = 640	154 (35.5); N = 434	1.05	0.81–1.36	0.715
BICLA at week 16	281 (43.8); N = 641	174 (40.0); N = 435	1.12	0.87–1.44	0.367
BICLA at week 20	281 (43.8); N = 641	170 (39.2); N = 434	1.15	0.89–1.48	0.282
BICLA at week 24	282 (44.1); N = 640	176 (40.6); N = 434	1.12	0.87–1.44	0.389
BICLA at week 28	282 (44.0); N = 641	170 (39.2); N = 434	1.21	0.94–1.56	0.141
BICLA at week 32	281 (43.8); N = 642	188 (43.3); N = 434	1.00	0.78–1.29	0.993
BICLA at week 36	280 (43.7); N = 641	185 (42.6); N = 434	1.01	0.79–1.30	0.942
BICLA at week 40	280 (43.8); N = 640	183 (42.1); N = 435	1.05	0.81–1.34	0.723
BICLA at week 44	281 (43.9); N = 640	188 (43.2); N = 435	1.00	0.78–1.29	0.973
BICLA at week 48	288 (44.9); N = 641	174 (40.0); N = 435	1.20	0.93–1.54	0.162
BICLA at week 52	297 (46.3); N = 641	177 (40.7); N = 435	1.20	0.94–1.55	0.146

Results from logistic regression analysis, adjusting for trial variance with BLISS-52 and BLISS-76 pooled as a reference. Data are presented as the number of events (percentage), odds ratio (OR), 95% confidence interval (CI), and *p* value. Statistically significant *p* values are in bold.

BEL: belimumab; BICLA: British Isles Lupus Assessment Group (BILAG)-Based Composite Lupus Assessment; CI: confidence interval; OR: odds ratio; PBO: placebo; SLEDAI-2K: Systemic Lupus Erythematosus Disease Activity Index 2000.

Supplemental Table S4. BICLA response from week 4 through week 52 in patients with positive anti-dsDNA levels at baseline treated with belimumab versus placebo.

	BEL; n (%); N	PBO; n (%); N	OR	95% CI	*p* value
**BICLA response**					
BICLA at week 4	326 (27.1); N=1204	181 (23.8); N=762	1.19	0.96–1.47	0.107
BICLA at week 8	420 (34.9); N=1204	217 (28.5); N=762	1.31	1.07–1.60	**0.009**
BICLA at week 12	467 (38.9); N=1202	241 (31.6); N=762	1.38	1.14–1.67	**0.001**
BICLA at week 16	528 (43.9); N=1203	255 (33.4); N=763	1.53	1.26–1.85	**<0.001**
BICLA at week 20	543 (45.1); N=1203	259 (33.9); N=763	1.57	1.30–1.90	**<0.001**
BICLA at week 24	581 (48.3); N=1202	273 (35.8); N=762	1.63	1.35–1.97	**<0.001**
BICLA at week 28	544 (45.2); N=1203	255 (33.5); N=762	1.63	1.35–1.97	**<0.001**
BICLA at week 32	557 (46.3); N=1204	266 (34.9); N=763	1.56	1.29–1.89	**<0.001**
BICLA at week 36	537 (44.6); N=1203	259 (34.0); N=762	1.53	1.26–1.85	**<0.001**
BICLA at week 40	557 (46.3); N=1202	251 (32.9); N=762	1.73	1.43–2.09	**<0.001**
BICLA at week 44	558 (46.4); N=1202	261 (34.2); N=763	1.63	1.35–1.97	**<0.001**
BICLA at week 48	543 (45.1); N=1203	252 (33.0); N=763	1.64	1.35–1.99	**<0.001**
BICLA at week 52	545 (45.3); N=1203	255 (33.5); N=762	1.59	1.31–1.92	**<0.001**

Results from logistic regression analysis, adjusting for trial variance with BLISS-52 and BLISS-76 pooled as a reference. Data are presented as the number of events (percentage), odds ratio (OR), 95% confidence interval (CI), and *p* value. Statistically significant *p* values are in bold.

Anti-dsDNA: anti double-stranded DNA antibodies; BEL: belimumab; BICLA: British Isles Lupus Assessment Group (BILAG)-Based Composite Lupus Assessment; CI: confidence interval; OR: odds ratio; PBO: placebo.

Supplemental Table S5. BICLA response from week 4 through week 52 in patients negative for anti-dsDNA at baseline treated with belimumab versus placebo.

	BEL; n (%); N	PBO; n (%); N	OR	95% CI	*p* value
**BICLA response**					
BICLA at week 4	97 (19.8); N=489	80 (23.1); N=346	0.82	0.58–1.15	0.245
BICLA at week 8	137 (28.0); N=489	88 (25.4); N=346	1.15	0.83–1.58	0.401
BICLA at week 12	168 (34.4); N=489	109 (31.6); N=346	1.08	0.80–1.45	0.634
BICLA at week 16	188 (38.4); N=489	118 (34.1); N=346	1.17	0.87–1.57	0.306
BICLA at week 20	190 (38.9); N=489	129 (37.4); N=346	1.01	0.76–1.36	0.937
BICLA at week 24	200 (40.9); N=489	128 (37.1); N=346	1.13	0.85–1.51	0.400
BICLA at week 28	200 (40.9); N=489	119 (34.5); N=346	1.26	0.94–1.69	0.124
BICLA at week 32	199 (40.7); N=489	126 (36.5); N=346	1.13	0.85–1.51	0.399
BICLA at week 36	198 (40.5); N=489	132 (38.3); N=346	1.05	0.79–1.40	0.731
BICLA at week 40	200 (40.9); N=489	136 (39.3); N=346	1.02	0.76–1.35	0.913
BICLA at week 44	200 (40.9); N=489	133 (38.4); N=346	1.10	0.82–1.46	0.524
BICLA at week 48	203 (41.5); N=489	130 (37.6); N=346	1.18	0.88–1.57	0.269
BICLA at week 52	209 (42.7); N=489	130 (37.6); N=346	1.22	0.92–1.63	0.172

Results from logistic regression analysis, adjusting for trial variance with BLISS-52 and BLISS-76 pooled as a reference. Data are presented as the number of events (percentage), odds ratio (OR), 95% confidence interval (CI), and *p* value. Statistically significant *p* values are in bold.

Anti-dsDNA: anti double-stranded DNA antibodies; BEL: belimumab; BICLA: British Isles Lupus Assessment Group (BILAG)-Based Composite Lupus Assessment; CI: confidence interval; OR: odds ratio; PBO: placebo.

Supplemental Table S6. BICLA response from week 4 through week 52 in patients with positive anti-dsDNA levels and/or low C3 and/or C4 levels at baseline treated with belimumab versus placebo.

	BEL; n (%)	PBO; n (%)	OR	95% CI	*p* value
**BICLA response**					
BICLA at week 4	346 (25.9); N=1335	203 (23.5); N=865	1.13	0.93–1.39	0.223
BICLA at week 8	460 (34.5); N=1335	244 (28.2); N=865	1.30	1.08–1.57	**0.006**
BICLA at week 12	516 (38.7); N=1333	272 (31.4); N=865	1.38	1.15–1.66	**0.001**
BICLA at week 16	580 (43.5); N=1334	292 (33.7); N=866	1.49	1.25–1.79	**<0.001**
BICLA at week 20	596 (44.7); N=1334	301 (34.8); N=866	1.49	1.25–1.78	**<0.001**
BICLA at week 24	638 (47.9); N=1333	311 (36.0); N=865	1.61	1.34–1.92	**<0.001**
BICLA at week 28	602 (45.1); N=1334	290 (33.5); N=865	1.62	1.35–1.94	**<0.001**
BICLA at week 32	612 (45.8); N=1335	302 (34.9); N=866	1.55	1.29–1.85	**<0.001**
BICLA at week 36	592 (44.4); N=1334	299 (34.6); N=865	1.48	1.24–1.77	**<0.001**
BICLA at week 40	609 (45.7); N=1333	293 (33.9); N=865	1.62	1.36–1.94	**<0.001**
BICLA at week 44	608 (45.6); N=1333	301 (34.8); N=866	1.55	1.30–1.86	**<0.001**
BICLA at week 48	597 (44.8); N=1334	292 (33.7); N=866	1.58	1.32–1.89	**<0.001**
BICLA at week 52	597 (44.8); N=1334	293 (33.9); N=865	1.54	1.29–1.85	**<0.001**

Results from logistic regression analysis, adjusting for trial variance with BLISS-52 and BLISS-76 pooled as a reference. Data are presented as the number of events (percentage), odds ratio (OR), 95% confidence interval (CI), and *p* value. Statistically significant *p* values are in bold.

Anti-dsDNA: anti double-stranded DNA antibodies; BEL: belimumab; BICLA: British Isles Lupus Assessment Group (BILAG)-Based Composite Lupus Assessment; CI: confidence interval; C3: complement component 3; C4: complement component 4; OR: odds ratio; PBO: placebo.

Supplemental Table S7. BICLA response from week 4 through week 52 in patients negative for anti-dsDNA and with normal/high C3 and C4 levels at baseline treated with belimumab versus placebo.

	BEL; n (%)	PBO; n (%)	OR	95% CI	*p* value
**BICLA response**					
BICLA at week 4	77 (21.5); N=358	58 (23.9); N=243	0.89	0.60–1.32	0.558
BICLA at week 8	97 (27.1); N=358	61 (25.1); N=243	1.11	0.76–1.63	0.581
BICLA at week 12	119 (33.2); N=358	78 (32.2); N=242	0.97	0.68–1.39	0.873
BICLA at week 16	136 (38.0); N=358	81 (33.3); N=243	1.13	0.80–1.61	0.483
BICLA at week 20	137 (38.3); N=358	87 (36.0); N=242	1.02	0.72–1.45	0.900
BICLA at week 24	143 (39.9); N=358	90 (37.2); N=242	1.03	0.73–1.46	0.857
BICLA at week 28	142 (39.7); N=358	84 (34.7); N=242	1.16	0.82–1.65	0.392
BICLA at week 32	144 (40.2); N=358	90 (37.2); N=242	1.03	0.73–1.46	0.855
BICLA at week 36	143 (39.9); N=358	92 (38.0); N=242	0.99	0.70–1.40	0.974
BICLA at week 40	148 (41.3); N=358	94 (38.7); N=243	1.02	0.72–1.44	0.912
BICLA at week 44	150 (41.9); N=358	93 (38.3); N=243	1.12	0.79–1.57	0.521
BICLA at week 48	149 (41.6); N=358	90 (37.0); N=243	1.17	0.83–1.65	0.377
BICLA at week 52	157 (43.9); N=358	92 (37.9); N=243	1.22	0.87–1.72	0.253

Results from logistic regression analysis, adjusting for trial variance with BLISS-52 and BLISS-76 pooled as a reference. Data are presented as the number of events (percentage), odds ratio (OR), 95% confidence interval (CI), and *p* value. Statistically significant *p* values are in bold.

Anti-dsDNA: anti double-stranded DNA antibodies; BEL: belimumab; BICLA: British Isles Lupus Assessment Group (BILAG)-Based Composite Lupus Assessment; CI: confidence interval; C3: complement component 3; C4: complement component; OR: odds ratio; PBO: placebo.

Supplemental Table S8. BICLA response from week 4 through week 52 in patients with a prednisone equivalent dose >7.5 mg/day at baseline treated with belimumab versus placebo.

	BEL; n (%)	PBO; n (%)	OR	95% CI	*p* value
**BICLA response**					
BICLA at week 4	283 (26.6); N=1064	160 (23.5); N=680	1.22	0.97–1.53	0.089
BICLA at week 8	365 (34.3); N=1064	203 (29.9); N=680	1.21	0.99–1.49	0.076
BICLA at week 12	408 (38.4); N=1063	223 (32.8); N=680	1.30	1.06–1.59	**0.013**
BICLA at week 16	458 (43.0); N=1064	231 (33.9); N=681	1.46	1.19–1.79	**<0.001**
BICLA at week 20	475 (44.7); N=1063	239 (35.1); N=681	1.48	1.21–1.80	**<0.001**
BICLA at week 24	499 (46.9); N=1063	253 (37.2); N=680	1.47	1.20–1.79	**<0.001**
BICLA at week 28	484 (45.5); N=1063	237 (34.9); N=680	1.52	1.25–1.86	**<0.001**
BICLA at week 32	481 (45.2); N=1064	246 (36.1); N=681	1.41	1.15–1.72	**0.001**
BICLA at week 36	465 (43.7); N=1063	251 (36.9); N=680	1.29	1.05–1.57	**0.014**
BICLA at week 40	483 (45.4); N=1063	245 (36.0); N=680	1.45	1.19–1.77	**<0.001**
BICLA at week 44	488 (45.9); N=1063	258 (37.9); N=681	1.37	1.12–1.67	**0.002**
BICLA at week 48	477 (44.8); N=1064	246 (36.1); N=681	1.42	1.16–1.73	**0.001**
BICLA at week 52	481 (45.2); N=1064	249 (36.6); N=680	1.39	1.14–1.70	**0.001**

Results from logistic regression analysis, adjusting for trial variance with BLISS-52 and BLISS-76 pooled as a reference. Data are presented as the number of events (percentage), odds ratio (OR), 95% confidence interval (CI), and *p* value. Statistically significant *p* values are in bold.

BEL: belimumab; BICLA: British Isles Lupus Assessment Group (BILAG)-Based Composite Lupus Assessment; CI: confidence interval; OR: odds ratio; PBO: placebo.

Supplemental Table S9. BICLA response from week 4 through week 52 in patients with a prednisone equivalent dose ≤7.5 mg/day at baseline treated with belimumab versus placebo.

	BEL; n (%)	PBO; n (%)	OR	95% CI	*p* value
**BICLA response**					
BICLA at week 4	140 (22.3); N=629	101 (23.6); N=428	0.87	0.64–1.17	0.343
BICLA at week 8	192 (30.5); N=629	102 (23.8); N=427	1.38	1.04–1.83	**0.027**
BICLA at week 12	227 (36.1); N=628	127 (29.7); N=428	1.27	0.97–1.66	0.083
BICLA at week 16	258 (41.1); N=628	142 (33.2); N=427	1.36	1.04–1.76	**0.022**
BICLA at week 20	258 (41.0); N=629	149 (34.9); N=427	1.25	1.96–1.62	0.098
BICLA at week 24	282 (44.9); N=628	148 (34.7); N=427	1.48	1.14–1.92	**0.003**
BICLA at week 28	260 (41.3); N=629	137 (32.1); N=427	1.52	1.16–1.97	**0.002**
BICLA at week 32	275 (43.7); N=629	146 (34.2); N=427	1.48	1.14–1.92	**0.003**
BICLA at week 36	270 (42.9); N=629	140 (32.8); N=427	1.53	1.18–1.99	**0.001**
BICLA at week 40	274 (43.6); N=628	142 (33.2); N=428	1.53	1.18–1.99	**0.001**
BICLA at week 44	270 (43.0); N=628	136 (31.8); N=428	1.62	1.25–2.11	**<0.001**
BICLA at week 48	269 (42.8); N=628	136 (31.8); N=428	1.62	1.25–2.11	**<0.001**
BICLA at week 52	273 (43.5); N=628	136 (31.8); N=428	1.64	1.26–2.13	**<0.001**

Results from logistic regression analysis, adjusting for trial variance with BLISS-52 and BLISS-76 pooled as a reference. Data are presented as the number of events (percentage), odds ratio (OR), 95% confidence interval (CI), and *p* value. Statistically significant *p* values are in bold.

BEL: belimumab; BICLA: British Isles Lupus Assessment Group (BILAG)-Based Composite Lupus Assessment; CI: confidence interval; OR: odds ratio; PBO: placebo.

Supplemental Table S10. Influence of concomitant antimalarial treatment on BICLA response at week 52.

	Group 1	Group 2	OR	95% CI	*p* value
**BICLA response**					
	BEL; n (%)	PBO; n (%)			
BICLA at week 52	213 (41.3); N=774	105 (31.3); N=335	1.25	0.95–1.64	0.0114
	PBO + AMA; n (%)	PBO; n (%)			
BICLA at week 52	280 (36.2); N=774	105 (31.3); N=335	1.51	1.13–2.02	**0.005**
	BEL + AMA; n (%)	PBO; n (%)			
BICLA at week 52	541 (46.0); N=1177	105 (31.3); N=335	1.82	1.40–2.37	**<0.001**
	BEL + AMA; n (%)	BEL; n (%)			
BICLA at week 52	541 (46.0); N=1177	213 (41.3); N=774	1.22	0.99–1.51	0.065

Results from univariable logistic regression analysis of BICLA response at week 52 in subgroups of patients stratified based on treatment arm and concomitant treatment with antimalarial agents. Data are presented as numbers, odds ratio (OR), 95% confidence interval (CI), and *p* value for each comparison. Statistically significant *p* values are in bold.

AMA: antimalarial agents; BEL: belimumab; BICLA: British Isles Lupus Assessment Group (BILAG)-Based Composite Lupus Assessment; CI: confidence interval; OR: odds ratio; PBO: placebo.

Supplemental Table S11. Factors associated with BICLA response at week 52 in patients with SLE in the pooled belimumab trial population.

	N	OR	95% CI	*p* value
**Demographics**				
Age	2802	1.00	0.99–1.00	0.501
Male sex	2802	1.04	0.75–1.44	0.806
Race (ref.: White/Caucasian)	2802			
Asian		1.17	0.92–1.49	0.206
Black/African American		0.76	0.54–1.08	0.127
Indigenous American		1.34	1.02–1.76	**0.036**
**Clinical Features**				
BMI at baseline	2707	0.99	0.98–1.00	0.131
SLE disease duration at baseline	2801	0.98	0.97–0.99	**0.001**
cSLEDAI-2K score at baseline	2802	0.78	0.66–0.91	**0.001**
SDI score ≥1 at baseline	2802	0.74	0.63–0.87	**<0.001**
SDI score ≥2 at baseline	2802	0.63	0.50–0.79	**<0.001**
**Serological status**				
Anti-dsDNA (+) at baseline	2802	0.98	0.83–1.15	0.770
Anti-Smith (+)				
at baseline	1808	0.78	0.63–0.95	**0.016**
at any measurement during follow-up	1810	0.77	0.62–0.94	**0.012**
Anti-RNP (+)				
at baseline	1204	0.68	0.53–0.87	**0.002**
at any measurement during follow-up	1207	0.67	0.52–0.87	**0.002**
Anti-ribosomal P (+)				
at baseline	2203	0.88	0.73–1.06	0.192
at any measurement during follow-up	2203	0.89	0.74–1.07	0.211
aPL (+) at baseline				
aCL				
IgA	2676	1.04	0.62–1.74	0.892
IgG	2680	0.75	0.59–0.95	**0.019**
IgM	2680	0.82	0.63–1.06	0.136
Anti-β2GPI				
IgA	1212	0.92	0.68–1.24	0.593
IgG	1212	1.40	0.69–2.82	0.354
IgM	1212	0.87	0.53–1.42	0.567
LAC	1197	1.13	0.83–1.53	0.432
aPL (+) at any measurement during follow-up	2684	0.89	0.75–1.06	0.190
BLyS/BAFF levels at baseline	2348	0.90	0.84–0.96	**0.001**

Low C3 at baseline	2802	0.80	0.68–0.94	**0.006**
Low C4 at baseline	2802	0.75	0.64–0.89	**0.001**
**Medications**				
Pred eq. dose >7.5 mg/day at baseline	2802	1.10	0.94–1.29	0.232
AMA at baseline	2802	1.21	1.03–1.43	**0.024**
Immunosuppressants at baseline	2802			
Azathioprine		0.89	0-73–1.08	0.247
Methotrexate		0.91	0.72–1.15	0.441
Mycophenolic acid		0.86	0.69–1.06	0.152
Cyclophosphamide		0.98	0.52–1.87	0.963
Tacrolimus		0.68	0.43–1.08	0.102
Ciclosporin		0.89	0.54–1.48	0.657
Leflunomide		0.73	0.44–1.20	0.211
BEL approved dose	2802	1.47	1.25–1.72	**<0.001**

Results from logistic regression analysis adjusting for trial variance with BLISS-52 and BLISS-76 pooled as a reference, with BICLA response at week 52 as the dependant variable. Data are presented as numbers, odds ratio (OR), 95% confidence interval (CI), and *p* value. Statistically significant *p* values are in bold.

(+): positive; aCL: anti-cardiolipin antibodies; AMA: antimalarial agents; anti-dsDNA: anti-double-stranded DNA antibodies; anti-RNP: anti-U1-ribonucleoprotein antibodies; anti-β2GPI: anti-β2 glycoprotein I antibodies; aPL: antiphospholipid antibodies; BEL: belimumab; BICLA: British Isles Lupus Assessment Group (BILAG)-Based Composite Lupus Assessment; BLyS/BAFF: B-Lymphocyte stimulator/ B cell activating factor belonging to the tumour necrosis factor family; BMI: body mass index; CI: confidence interval; cSLEDAI-2K: clinical Systemic Lupus Erythematosus Disease Activity Index 2000; C3: complement component 3; C4: complement component 4; eq.: equivalent; Ig: immunoglobulin; LAC: lupus anticoagulant; OR: odds ratio; Pred.: prednisone; SDI: Systemic Lupus International Collaborating Clinics (SLICC)/American College of Rheumatology (ACR) Damage Index; SLE: systemic lupus erythematosus.

Supplemental Table S12. Factors associated with BICLA response at week 52 in patients with SLE in the pooled belimumab trial population by multivariable analysis.

	OR	95% CI	*p* value
**Demographics**			
Age	1.00	0.99–1.01	0.868
Male sex	1.08	0.77–1.51	0.664
Race (ref.: White/Caucasian)			
Asian	1.18	0.91–1.51	0.209
Black/African American	0.79	0.56–1.13	0.196
Indigenous American	1.30	0.98–1.73	0.064
**Clinical Features**			
SLE disease duration at baseline	0.99	0.97–1.00	0.077
cSLEDAI-2K score at baseline	0.83	0.70–0.98	**0.030**
SDI >2 score at baseline	0.68	0.53–0.88	**0.003**
**Serological status**			
aCL IgG (+) at baseline	0.84	0.65–1.08	0.163
Low C3 at baseline	0.90	0.74–1.09	0.265
Low C4 at baseline	0.80	0.65–0.97	**0.025**
**Medications**			
AMA at baseline	1.15	0.97–1.38	0.112
BEL approved dose	1.45	1.23–1.71	**<0.001**

Results from multivariable logistic regression analysis adjusting for trial variance with BLISS-52 and BLISS-76 pooled as a reference, with BICLA response at week 52 as the dependant variable. Data are presented as the odds ratio (OR), 95% confidence interval (CI), and *p* value. Statistically significant *p* values are in bold. The total number of patients for this analysis was 2680.

(+): positive; aCL: anticardiolipin antibodies; AMA: antimalarials; BEL: belimumab; BICLA: British Isles Lupus Assessment Group (BILAG)-Based Composite Lupus Assessment; CI: confidence interval; cSLEDAI-2K: clinical Systemic Lupus Erythematosus Disease Activity Index 2000; C3: complement component 3; C4: complement component 4; eq.: equivalent; IgG: immunoglobulin G; OR: odds ratio; Pred.: prednisone; SDI: Systemic Lupus International Collaborating Clinics (SLICC)/American College of Rheumatology (ACR) Damage Index; SLE: systemic lupus erythematosus.
